# Upregulation of Nrf2 in myocardial infarction and ischemia-reperfusion injury of the heart

**DOI:** 10.1371/journal.pone.0299503

**Published:** 2024-03-15

**Authors:** Sahar Zuberi, Hira Rafi, Azhar Hussain, Satwat Hashmi

**Affiliations:** 1 Department of Biological and Biomedical Sciences, Aga Khan University, Karachi Pakistan; 2 Department of Physiology, Rashid Latif Khan University Medical College, Lahore, Pakistan; 3 Postdoctoral Fellow Northwestern University Feinberg School of Medicine Chicago, Illinois, United States of America; National Institutes of Health, UNITED STATES

## Abstract

Myocardial infarction (MI) is a leading cause of morbidity and mortality in the world and is characterized by ischemic necrosis of an area of the myocardium permanently devoid of blood supply. During reperfusion, reactive oxygen species are released and this causes further insult to the myocardium, resulting in ischemia-reperfusion (IR) injury. Since Nrf2 is a key regulator of redox balance, it is essential to determine its contribution to these two disease processes. Conventionally Nrf2 levels have been shown to rise immediately after ischemia and reperfusion but its contribution to disease process a week after the injury remains uncertain. Mice were divided into MI, IR injury, and sham surgery groups and were sacrificed 1 week after surgery. Infarct was visualized using H&E and trichrome staining and expression of Nrf2 was assessed using immunohistochemistry, Western blot, and ELISA. MI displayed a higher infarct size than the IR group (MI: 31.02 ± 1.45%, IR: 13.03 ± 2.57%; p < 0.01). We observed a significantly higher expression of Nrf2 in the MI group compared to the IR model using immunohistochemistry, spot densitometry of Western blot (MI: 2.22 ± 0.16, IR: 1.81 ± 0.10, Sham: 1.52 ± 0.13; p = 0.001) and ELISA (MI: 80.78 ± 27.08, IR: 31.97 ± 4.35; p < 0.01). There is a significantly higher expression of Nrf2 in MI compared to the IR injury group. Modulation of Nrf2 could be a potential target for therapeutics in the future, and its role in cardioprotection can be further investigated.

## 1. Introduction

Coronary heart disease (CHD) results in a high burden of morbidity and mortality in individuals above the age of 35 years, accounting for 12.7% of deaths worldwide [1, 2]. Due to advancements in healthcare, mortality due to CHD has improved by 50% in high-income countries in the last decade. Unfortunately, in low and middle-income regions, it still accounts for 80% of the deaths [2].

Myocardial infarction has an incidence of 1,655 per 100,000 individuals (1.72% of the population of the world) [3]. Timely treatment by percutaneous coronary intervention has drastically improved survival in acute setting, shifting the burden of morbidity to ischemia-reperfusion injury that follows.

Myocardial ischemia-reperfusion injury ensues when the decreased blood supply to the heart due to blockage of coronary vasculature is restored, resulting in further damage to the myocardium [4]. Mechanism of cell death includes a burst of release of reactive oxygen species which initiates oxidative stress. Furthermore, a rapid correction of acidosis results in mitochondrial injury calcium overload causes mitochondrial dysfunction and there is initiation of inflammation [5–8].

Nuclear factor (erythroid-derived 2)-related factor 2 (Nrf2) is a transcription factor that is not present at rest in the heart. It is expressed either during embryogenesis or when there is oxidative stress or ischemia/reperfusion injury [9]. Under resting conditions, it is ubiquitinated and subsequently degraded in the cytoplasm, with resultant minimal expression [10]. In event of oxidative stress, Nrf2 pathway is activated. Nrf2 translocates to the nucleus where it transcribes antioxidants [11, 12].

Nrf2 has been shown to have a cardioprotective role in diseases of the heart and is a key regulator of antioxidant defenses following myocardial infarction and ischemia-reperfusion injury. When there is ischemia-reperfusion injury of the heart, Nrf2 is activated. This results in a decrease in oxidative stress, thereby reducing apoptosis, alleviating mitochondrial damage and endoplasmic reticulum stress, suppressing inflammation and preventing pathological remodeling of the heart by boosting autophagy of misfolded proteins [11, 13, 14]. Nrf2 is activated two hours after ischemia-reperfusion injury and peaks at 24hours in the brain [15], however its time dependent activation in the heart remains unclear.

Oxidative stress has been reported to be maximally evident from day 3 to day 7 following ischemic insult to the heart [16]. Since Nrf2 is an antioxidant, we expect its levels to also be elevated during this time. The significance of Nrf2 in the first few days following ischemic insult is suggestive of its important role in pathogenesis, however, there is scarcity of literature reporting its activity a week following the ischemic injury where oxidative stress is still at its peak [16, 17].

Thus, the objective of this study was to determine the levels of Nrf2 one week post ischemic injury when the oxidative stress is still greatly evident.

## 2. Methods

12–14 week old BALB/c male mice weighing between 20-30g were obtained from Aga Khan Hospital animal facility and acclimatized for 3 days with chow diet and tap water *ad libitum* at 25± C temperature and 12:12 hour light/dark cycle. Protocol was approved from institutional Ethics Committee for Animal Care & Use (97-ECACU-BBS-21) and mice (n = 48) were divided into three groups (n = 8 in each group): MI, IR and sham experimental surgery models. All animal surgeries were performed under anesthesia by an investigator who had previous experience in setting up the in-vivo model and had completed courses in animal sciences and laboratory safety. We ensured there was minimal animal suffering by daily monitoring of animals for any signs of pain or distress.

### 2.1 Animal model of ischemia-reperfusion injury

Anesthesia was achieved by 4% isoflurane induction and maintained at 1% throughout the experiment. Mice were placed on a temperature-controlled heating pad, intubated using a 22-guage cannula and attached to a ventilator (Harvard apparatus- VentElite Small Animal Ventilator). Eyes were lubricated and rectal temperature was monitored.

The method for murine experimental IR and MI injury followed has been used elsewhere [18]. An incision was made on the skin in the 4^th^ intercostal space on the left, muscles were separated and, and chest was retracted to expose the left anterior descending artery (LAD). LAD was then ligated permanently using an 8–0 silk suture. In the IR model, Polyethylene (PE) tubing (0.5mm) was placed on left anterior descending artery (LAD) and it was temporarily ligated and at 30mins time point, the knot on top of the PE tube was cut. Ischemia was confirmed by blanching of the left ventricle. In sham operated mice, suture was passed under LAD but the artery was not ligated.

Thereafter, third and fourth ribs were approximated, muscles were restored to their original position and skin was closed using 4–0 proline. Opioid analgesic, Buprenorphine 0.1 mg/kg was administered subcutaneously for pain relief and the animals were monitored in an oxygen enriched setup till they regained consciousness. During first few days, moistened food was placed inside the cage to facilitate feeding. Animals were monitored daily to assess adequate mobility, grooming, and eating habits. In the circumstances that an animal displays signs of pain or distress such as excessive lethargy, hypoventilation/gasping, reclusive behavior or more than 10% weight loss, early euthanasia was to be employed. However, none of the animals was found in distress and hence no early euthanasia was performed.

At seven day time point post-procedure, mice were euthanized with lethal dose of intraperitoneal Ketamine (80mg/kg) and Xylazine (12mg/kg) and heart and serum samples were collected for further analysis.

### 2.2 Morphometric study

Difference in weight before performing experimental surgery and at the time of sacrifice was noted to assess weight gain or weight loss. Ratio of weight of left ventricle and total body weight at the time of sacrifice was also recorded.

#### Tissue processing

Heart samples were washed in cold PBS, weighed, fixed in 10% formalin and 1mm sections were cut in the coronal plane. The sections were dehydrated with increasing gradations of ethanol followed by immersion in xylene and embedding in paraffin.

### 2.3 Histopathology

3μm sections were prepared from the paraffin blocks using a rotary microtome (PFM rotary 3005 E, Germany). These sections were stained using hematoxylin and eosin (H&E). Sections were dewaxed using xylene and hydrated with decremental gradations of alcohol. After rinsing in tap water, the slides were placed in hematoxylin followed by eosin. Slides were then washed again with tap water and hydrated in incremental gradations of ethanol, cleared using xylene and mounted with DPX (06522, Sigma-Aldrich, Germany).

### 2.4 Modified masson’s trichrome stain for estimation of infarct size

Microwave procedure for Masson’s trichrome kit (HT15-1KT, Sigma-Aldrich, Germany) was used for infarct size estimation with the omission of counter staining with hematoxylin. Skin was the positive control.

5μm slides were prepared from paraffin blocks and mounted on Poly L-lysine coated slides. These slides were dewaxed in xylene and hydrated with decreasing gradations of alcohol and placed in a Coplin jar filled with Bouin’s solution (500ml distilled water was saturated with picric acid and then filtered 167ml formaldehyde and 33.3ml glacial acetic acid were then added to it). This was microwaved for 15seconds followed by a 5mins incubation in fume hood. Slides were then washed with tap water until yellow color on the slides disappeared. Slides were then stained with Biebrich Scarlet-Acid Fuchsin and microwaved for 12seconds, followed by a two minute incubation and then placed in Phosphotungstic/Phosphomolybdic acid (10ml Phosphotungstic acid and 10ml Phosphomolybdic acid is mixed in 20ml deionized water) and microwaved for 12seconds followed by 5mins incubation. Slides were then placed in Analine Blue solution, microwaved for nine seconds, followed by a one minute incubation. After several washes of deionized water, slides were placed in 1% acetic acid (1N acetic acid) for 45 seconds and washed with deionized water for two mins. This was followed by sequential dehydration with alcohol and clearing with two changes of xylene. Slides were then mounted with DPX.

#### Planimetry

Images that were taken under a microscope on 1.25x magnification. They were edited for brightness and contrast using ImageJ software and right ventricle was removed. Images of left ventricle were analyzed on ImageJ and MIQuant softwares and infarct size was calculated using the principle of color thresholding. Infarct size calculation in MIQuant software is automated. To calculate infarct size using ImageJ software, the following formula was used:

$$\text{Infarct}\;\text{size}=\frac{1-(\sum \;\text{A}/\sum \;\text{TA})}{100}$$

Σ A = sum of areas of normal tissue of all the ventricles and apex

Σ TA = sum of total areas of tissues of all the ventricles and apex

### 2.5 Immunohistochemistry

Immunohistochemistry was performed using a kit (GV82511-2CN EnVision FLEX DAB+ Substrate Chromogen System (Dako Omnis))

5μm sections were prepared from paraffin embedded cassettes and mounted on poly-L-lysine coated slides. These slides were heated in a mechanical oven for 1 hour and then dewaxed in xylene and sequentially hydrated with decreasing gradations of alcohol. For antigen retrieval, these slides were placed in preheated antigen retrieval buffer (DAKO antigen retrieval buffer prepared with TBST) in a water bath for one hour. After cooling and washing slides with TBST, sections were blocked using 3% peroxide for 30mins followed by protein block with 10% goat serum for 30mins. Tissue was then incubated for two hours with Nrf2 antibody (Polyclonal rabbit anti-mouse antibody 1:100 Cloud Clone, USA) followed by application of secondary antibody (EnVisionTM Detection System, DAKO, Agilent, USA) for 45mins. The slides were then washed with TBST and subsequently with distilled water followed by application of DAB Chromogen (EnVisionTM Detection System, DAKO, Agilent, USA) for 3mins. Next slides were washed and counterstained with hematoxylin. Tissue was then placed increasing gradations of ethanol followed by immersion in xylene and mounted using DPX. Mouse lung served as positive control and slides devoid of primary antibody served as negative controls.

Five slides of each group were analyzed using Image J using color thresholding technique.

### 2.6 Western blot

Extracted mouse heart samples were thawed and cleaned with cold saline. Left ventricle (LV) was incised out, weighed and homogenized with KCl lysis buffer (0.24g Tis-base, 1.64g NaCl, 1.64g KCl, 0.08g EDTA, 1ml Triton X-100, 1g Sodium deoxycholate and 2ml protease inhibitor diluted in distilled water) on ice bath. Sample was centrifuged at 14,000rpm, and supernatant was collected and stored at -80 °C. Protein content was measured using BCA protein assay method (Thermo Scientific Pierce BCA Protein Assay Kit).

25μg proteins were separated using 10% SDS gel and transferred onto a PVDF membrane at 60V. Membranes were blocked using 5% milk in TBST, followed by incubation at 4°C overnight with primary antibodies against NRF2 (Cloud Clone, USA). Membrane was then washed with TBST and then incubated with horseradish peroxidase (HRP)- linked secondary antibody (Cell Signaling, USA) for 1.5hour and developed using ECL kit (GE Healthcare Prime Western Blotting Detection Reagent 28980926). Results were visualized on chemidock and quantified using GelQuant (GelQuant.NET BiochemLabSolutions, USA) [19–21].

### 2.7 ELISA

Quantitative expression of Nrf2 was measured in tissue samples of the left ventricle of the heart homogenized in KCl lysis buffer using Nrf2 sandwich enzyme-linked immunosorbent assay according to manufacturer’s instructions (Nrf2, Cloud-Clone Corp., Houston, USA).

### 2.8 Statistical analysis

Data were analyzed on SPSS software version 23 and Microsoft Excel. Quantitative samples were compared using One-way ANOVA followed by post hoc analysis using Bonferroni’s and were described as mean ± standard deviation. P-value<0.05 was considered significant throughout the study.

## 3. Results

### 3.1 Morphometric and Morphological changes comparisons in Myocardial Infarction and in Ischemia-Reperfusion Injury

**Fig 1:**
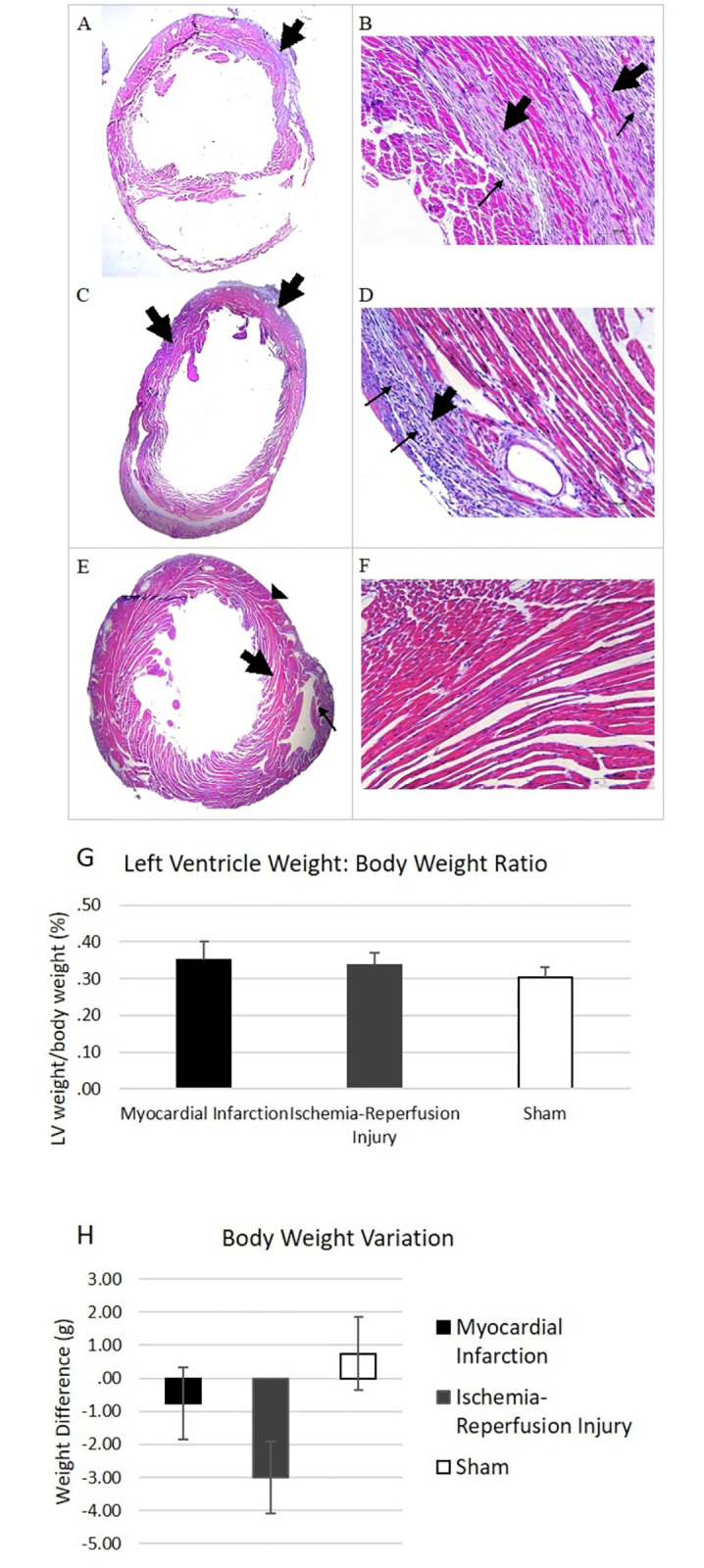
Morphometric analysis and visualization of infarcted area in the myocardium. Representative H&E images of the heart after seven days following sham, myocardial infarction (MI) or ischemia and reperfusion (IR) surgeries, in lower (A, C, E, 1.25x magnification) and higher (B, D, F, 20x magnification) and morphometric analysis of mice. MI (A,B) hearts shows dilatation of the left ventricle cavity and thinning of walls (A, thick arrow) and in high-power view, numerous inflammatory cells (B, thin arrow) and fibrotic tissue (collagen) deposition at the site of the infarct (B, thick arrow). On IR hearts (C,D), it’s shown dilatation of the left ventricle cavity and pronounced thinning of walls (C, thick arrow), and the presence of more abundant inflammatory cells (D, thin arrow) and fibrotic tissue (collagen) deposition at the site of the infarct (D, thick arrow). On sham hearts (E,F) it’s shown left ventricle (E, arrowhead), interventricular septum (E, thick arrow), and right ventricle arrow (E, thin arrow). Morphometric analysis of left ventricle weight/body weight (G) and weight difference before experimental surgery and at the time of sacrifice are not statistically significant.

Left ventricles of mice: total body weight ratio was comparable in all three experimental groups (MI: 0.35 ± 0.05g, IR: 0.34 ± 0.03g, Sham: 0.30 ± 0.03g, (p = 0.225 MI versus sham, p = 0.484 IR versus sham, p = 0.824 MI versus IR)). There was no significant difference between the body weight of mice before procedure and at the time of sacrifice either (MI: -0.75 ± 2.75g, IR: -3.00 ± 3.37g, Sham: 0.75 ± 1.26g, (p = 0.706 MI versus sham, p = 0.161 IR versus sham, p = 0.473 MI versus IR)) (Fig 1G and 1H).

H&E staining of mice that underwent IR surgery showed dilation of left ventricle with thinned out walls. There was infiltration of numerous inflammatory cells and deposition of collagen at the infarct site. Images were taken at 1.25x and 20x magnification. (Fig 1A–1F).

### 3.2 Increased infarct size in Myocardial Infarction as compared to Ischemia-Reperfusion Injury Model

**Fig 2:**
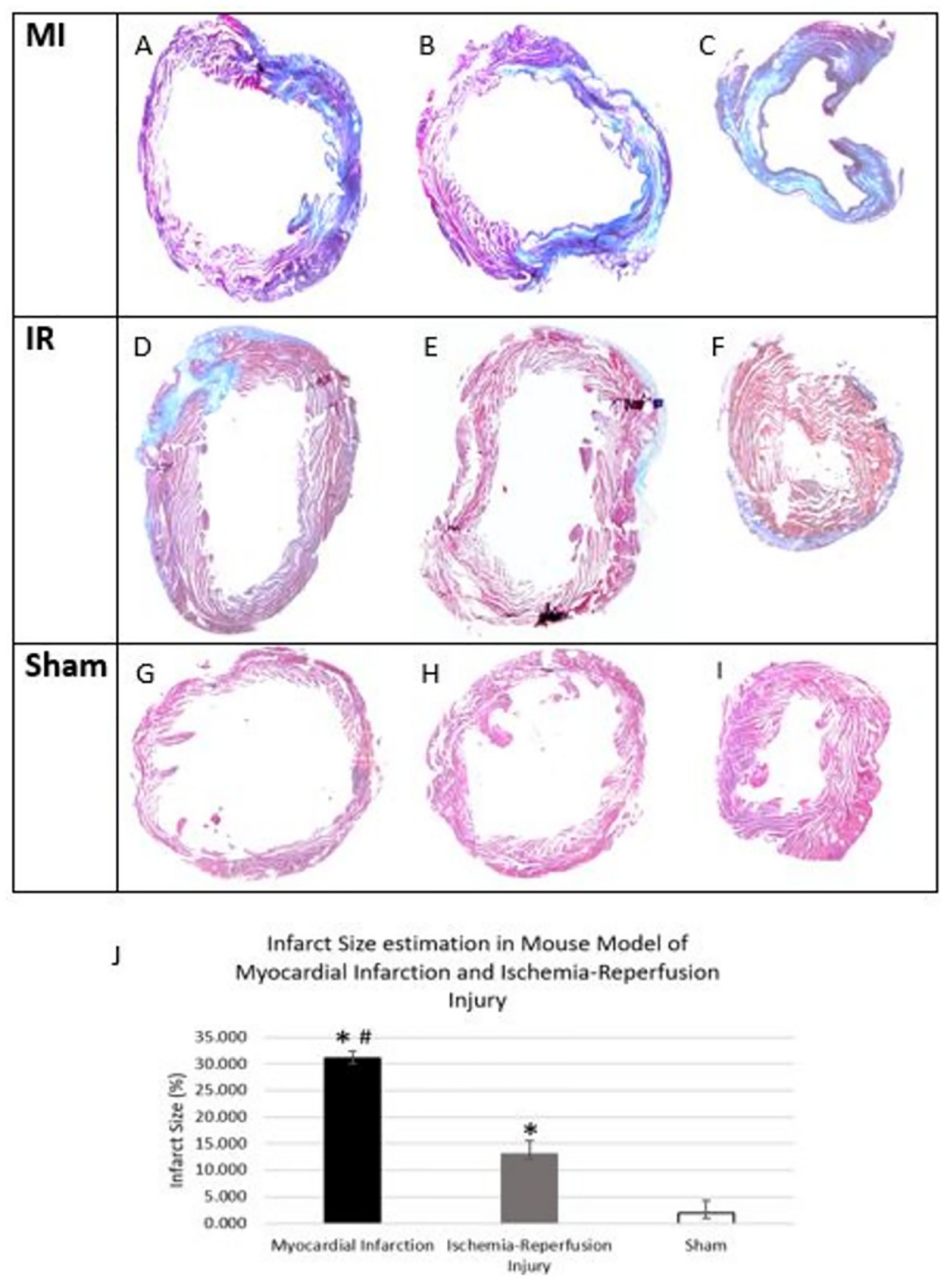
Masson’s trichrome stained representative images of animals that underwent MI, IR surgery and Sham and quantitative analysis of infarct sizes in these groups. The blue color indicates myocardial injury with increased collagen deposition (fibrosis). Images were taken at 1.25x by light microscope. (A,D,G: upper sections of the left ventricle, B,E,H: middle sections of the left ventricle, C,F,I: apex of the heart, J: Comparison of infarct sizes seven days after in MI and IR injury (n = 3 for each group) * p < 0.05 (versus sham), # p = 0.001 (MI versus IR).

**Fig 3:**
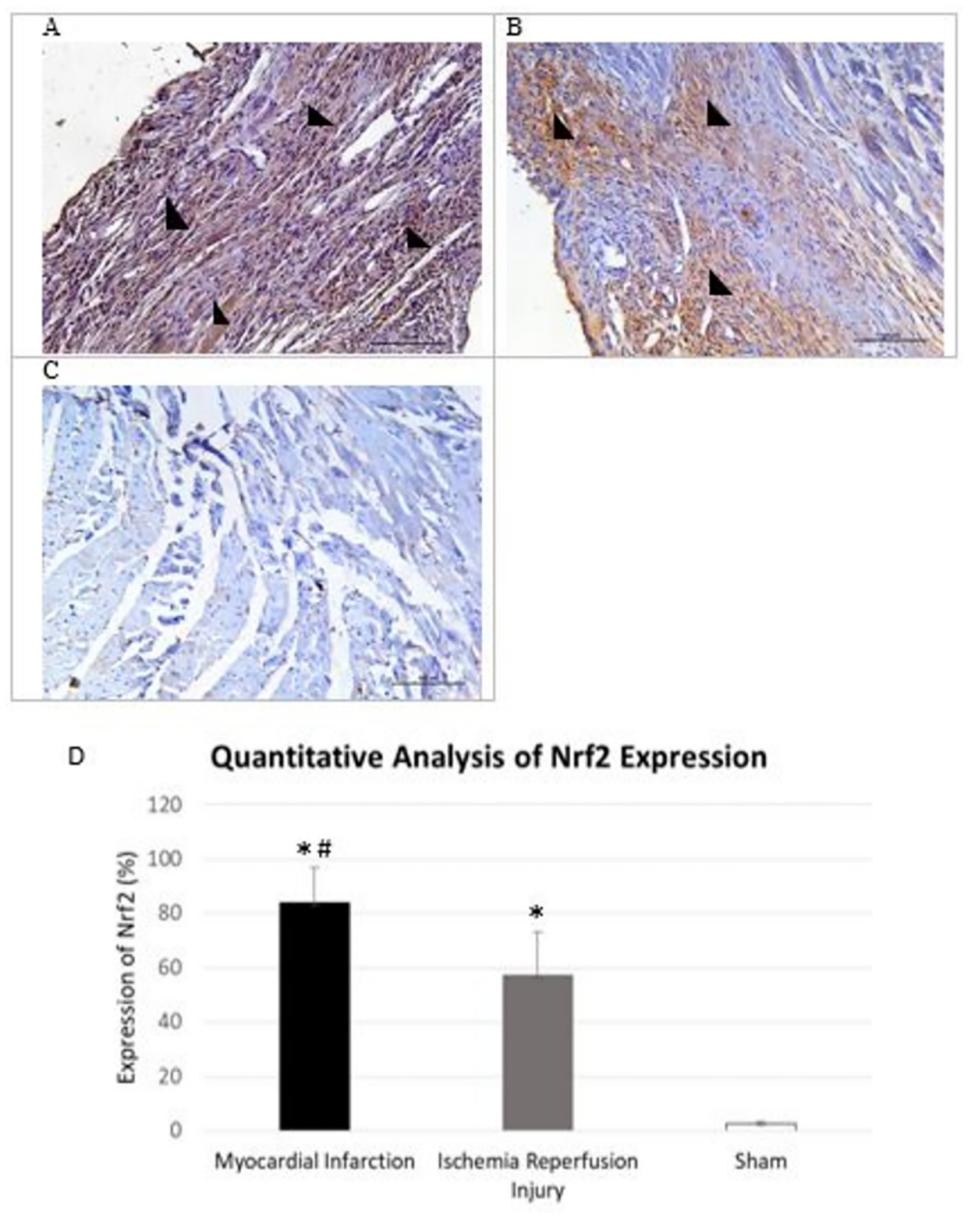
IHC images of Nrf2 in animals that underwent MI, IR injury and sham. Representative images of Nrf2 expression in the heart after seven days following sham, myocardial infarction (MI) or ischemia and reperfusion (IR) surgeries at 20x magnification, Streptavidin- biotin immunoperoxidase method. A; MI hearts show a much higher expression of Nrf2 in the wall of the left ventricle (black arrow head) especially at the site of injury, B; IR hearts show cytoplasmic expression of Nrf2 in the area of the IR injury (arrow heads). C; Sham operated heart shows faint cytoplasmic Nrf2 staining in the left ventricle and interventricular septum (arrow heads), D; Quantitative analysis of Nrf2 images (n = 5 in each group) shows a significantly high expression of Nrf2 in MI and IR compared to Sham. *p<0.05 (versus sham), #p<0.05 (MI versus IR).

Successful MI and IR surgery was determined by visualizing infarct area using Modified Masson’s trichrome staining. Trichrome staining displayed a significantly higher infarcted area (blue staining) in the myocardial infarction model (Fig 2A–2C) than in ischemia-reperfusion injury model (Fig 2D–2F) and sham operated group (Fig 2G–2I). Planimetry using ImageJ software showed a higher infarct size in MI group compared to IR injury group. (MI: 31.0±1.5%, IR:13.0±2.6%, p = 0.001). The infarct size in both these groups was significantly higher than the sham group (MI: 31.0±1.5%, Sham: 2.0±2.3%; p<0.001) (IR:13.0±2.6%, Sham: 2.0±2.3%; p = 0.005) (Fig 3).

### 3.3 Nrf2 has a higher expression in Myocardial Infarction and in Ischemia-Reperfusion Injury compared to Sham

**Fig 4:**
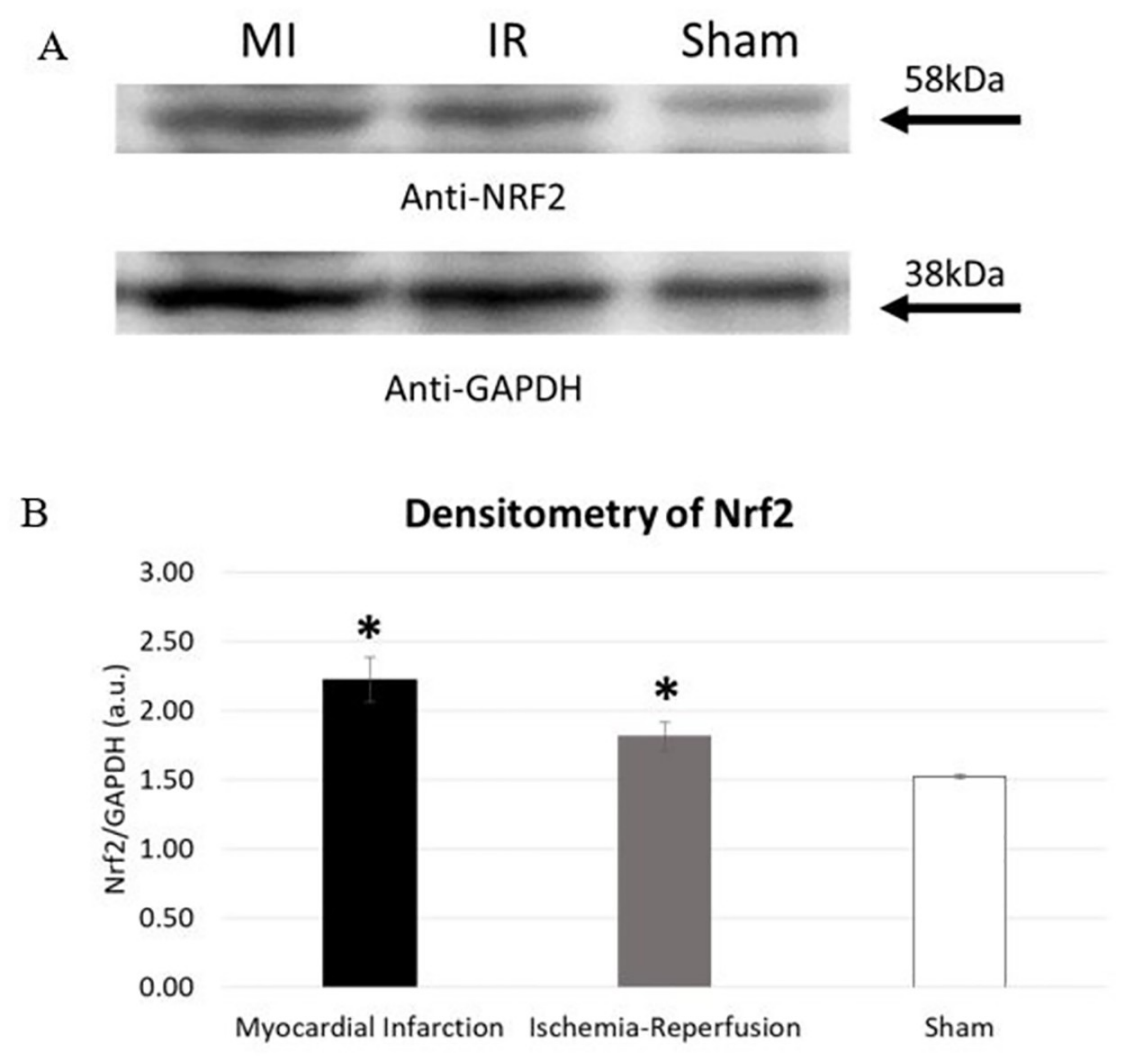
Upregulation of Nrf2 in MI and IR injury compared to Sham. A = upregulation of Nrf2 in MI compared to sham, B = Spot densitometry shows increased expression of Nrf2 in MI group compared to Sham. a.u. = arbitrary units (n = 3), *p = <0.05 (versus sham), p<0.05 (MI versus IR).

**Fig 5:**
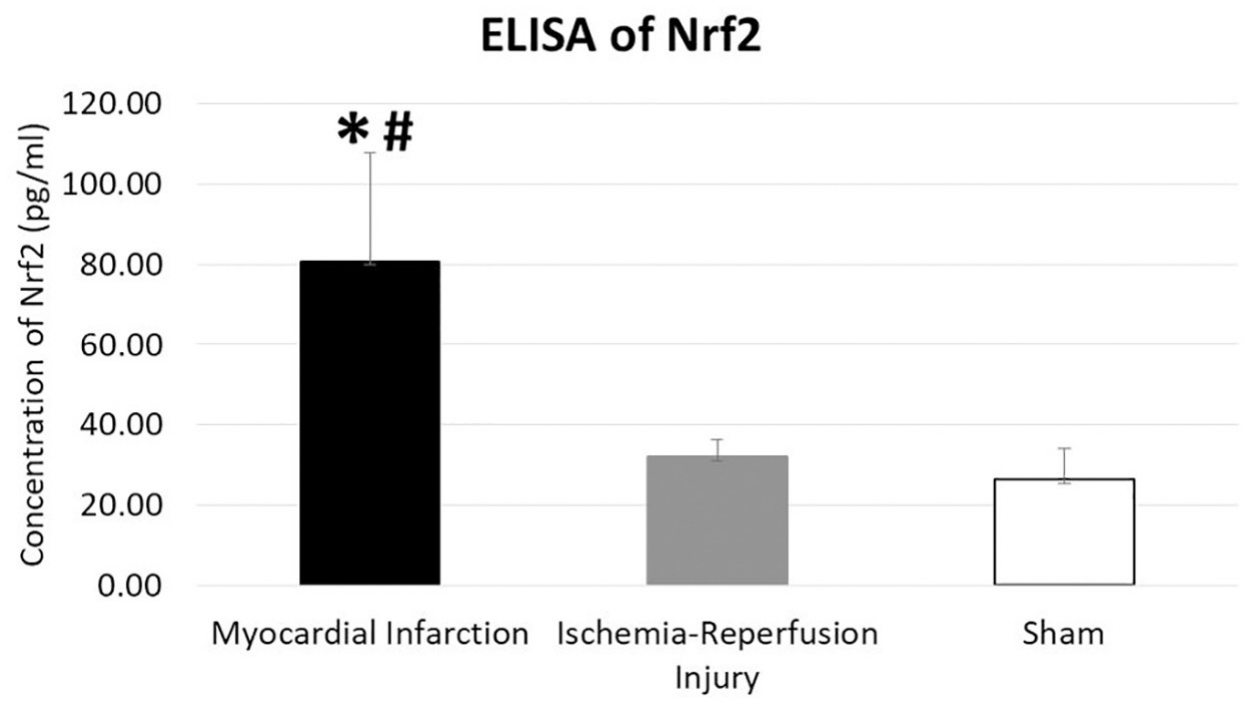
ELISA of Nrf2 demonstrating a higher expression in the MI group. *p = 0.001(versus sham), #p = 0.001 (MI versus IR).

IHC results shows an increased expression of Nrf2 at the infarct site in mice that underwent MI (Fig 3A) compared to IR injury group (Fig 3B). Nrf2 antibody stained minimally in the sham operated counterparts (Fig 3C). Quantitative analysis of IHC images showed a statistically significant increase in expression of Nrf2 in MI and IR compared to sham (MI: 83.9 ± 12.8%, IR: 57.1 ± 16.1%, sham: 2.8 ± 0.8% (p<0.001 MI versus sham, p = 0.002 IR versus sham, p = 0.001 MI versus IR)). Western blot also exhibited an upregulation of Nrf2 in MI and IR injury group compared to sham (Fig 4). The bands were quantified using spot densitometry normalized using GAPDH. Spot densitometry showed a significantly higher expression of Nrf2 in MI group compared to IR group and sham operated mice (MI: 2.2 ± 0.2, IR: 1.8 ± 0.1, Sham: 1.5 ± 0.1 (p = 0.017 MI versus sham, p = 0.037 IR versus sham, p = 0.028 MI versus IR) (Fig 4B). Nrf2 was further quantified using ELISA and that yielded the same result (MI: 80.8 ± 27.1pg/ml, IR: 32.0 ± 4.4pg/ml, Sham: 26.4 ± 7.8pg/ml; (p = 0.001 MI versus sham, p = 0.205 IR versus sham, p = 0.001 MI versus IR) (Fig 5).

## 4. Discussion

Murine model of ischemia-reperfusion injury and myocardial infarction was employed to study the levels of Nrf2 in these two models. The infarct size noted in MI (31.0±1.5%) and IR (19.5±6.3%) are consistent with existing literature (MI: 28–50%; IR with 30mins ligation of LAD: 12.23–30%); thus establishing the reliability of the mouse models [22].

In keeping with literature, we observed no significant difference in change in body weight before experimental surgery and 1 week following surgery [23]. Yang F *et al* 2002 reported that MI hearts weigh more than sham. Although the MI hearts in our study weighed more than sham hearts, the difference was not statistically significant [24].

Nrf2 is not expressed in a healthy heart [25]; it gets activated when the heart undergoes oxidative stress [25]. Although the role of Nrf2 in conjunction with oxidative stress in ischemia-reperfusion injury of the heart has been explored, albeit whether Nrf2 is cardioprotective or not remains unclear; leaving room for further investigation of its role in myocardial Infarction [26].

Conventionally, Nrf2 was considered cardioprotective but lately there seems to be a disparity in this statement and its ‘dark side’ is also being explored [27]. A few studies have demonstrated a reduction in the degree of cardioprotection and an increase in infarct size and rapid progression to heart failure in the days following ischemia-reperfusion injury in Nrf2KO mice [26, 28, 29]; as opposed to improvement in cardiac function in Nrf2KO mice reported by Erkens *et al* 2018 [30]. Qin Q *et al* 2015 addressed these discrepancies by studying the role of Nrf2 in autophagy and concluded that when autophagy is intact, Nrf2 is cardio-protective as opposed to when autophagy is impaired where Nrf2 leads to cardiac failure by initiating maladaptive cardiac remodeling [31]. These studies suggest Nrf2 is a key player in the pathogenesis of MI and IR.

We report a much higher level of Nrf2 in murine MI and IR model when compared to sham operated mice a week after surgery, thus suggestive of a role of Nrf2 in pathogenesis of MI and IR a week following injury, which also corresponds to the time when oxidative stress is maximal [16]. Stramer B *et al* 2007 identified macrophages as the predominant cell type in this phase [32]. This, coupled with the discovery by Zhang *et al* 2021 that Nrf2 works through recruitment of macrophages, explains the why Nrf2 gets activated at seven day time point [31, 33]. Since there is more pronounced inflammation in MI compared to IR model, the expression of Nrf2 is also higher in the MI model [18].

Furthermore, a greater infarct size would also be expected to result in more oxidative damage in MI compared to IR injury [22]. Since Nrf2 is an antioxidant, higher oxidative stress would amount to a higher surge of Nrf2 such as in MI. Subsequently myofibroblasts are active 4–7 days after ischemic injury and macrophages are also maximally activated seven days following ischemic insult, this makes one week time point ideal to study macrophage-fibroblast transition to cardiac fibrosis and the complications that result from it [32, 34].

In conclusion, the expression of Nrf2 is higher in the permanent ligation myocardial infarction model when compared to temporary ligation ischemia-reperfusion injury model seven days after surgical insult to the myocardium. This corresponds to the time when oxidative stress is the greatest and fibroblasts and macrophages are also maximally activated. Since Nrf2 is elevated in MI and IR model, its effect on pathogenesis, especially the alterations that are linked to oxidative stress, can be studied further by modulating its level and observing the effects that ensue.

## Supporting information

S1 Data (XLSX)

S1 Raw images (PDF)
