# Supplementary figures and images for: Upregulation of Nrf2 in myocardial infarction and ischemia-reperfusion injury of the heart
Source: PLoS One. 2024 Mar 15;19(3):e0299503. doi: 10.1371/journal.pone.0299503 (PMC10942075; doi:10.1371/journal.pone.0299503)

MI sham IR X

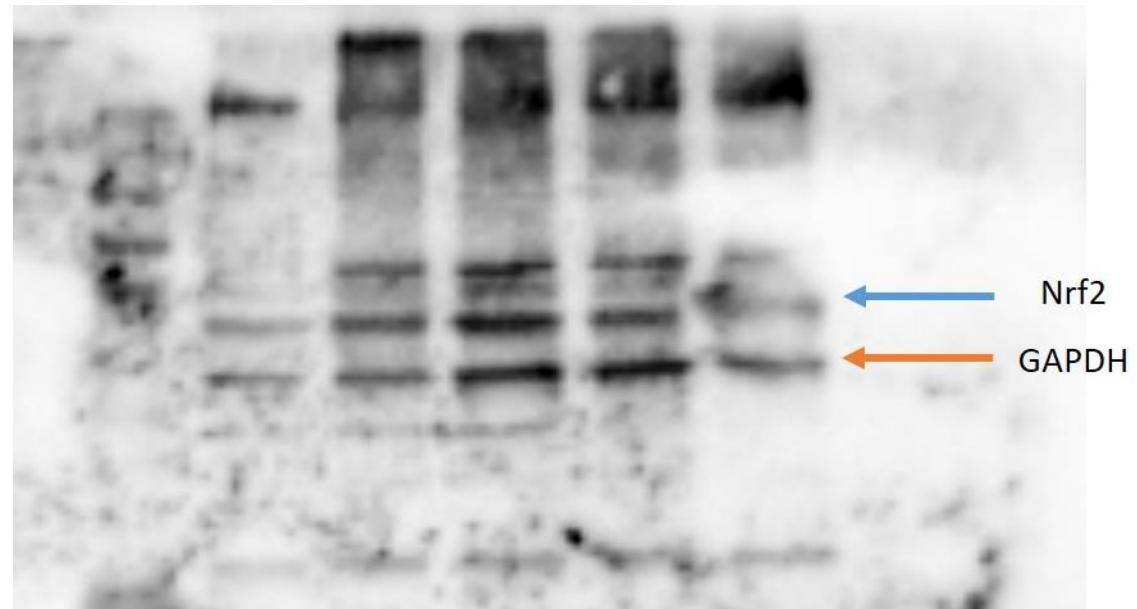

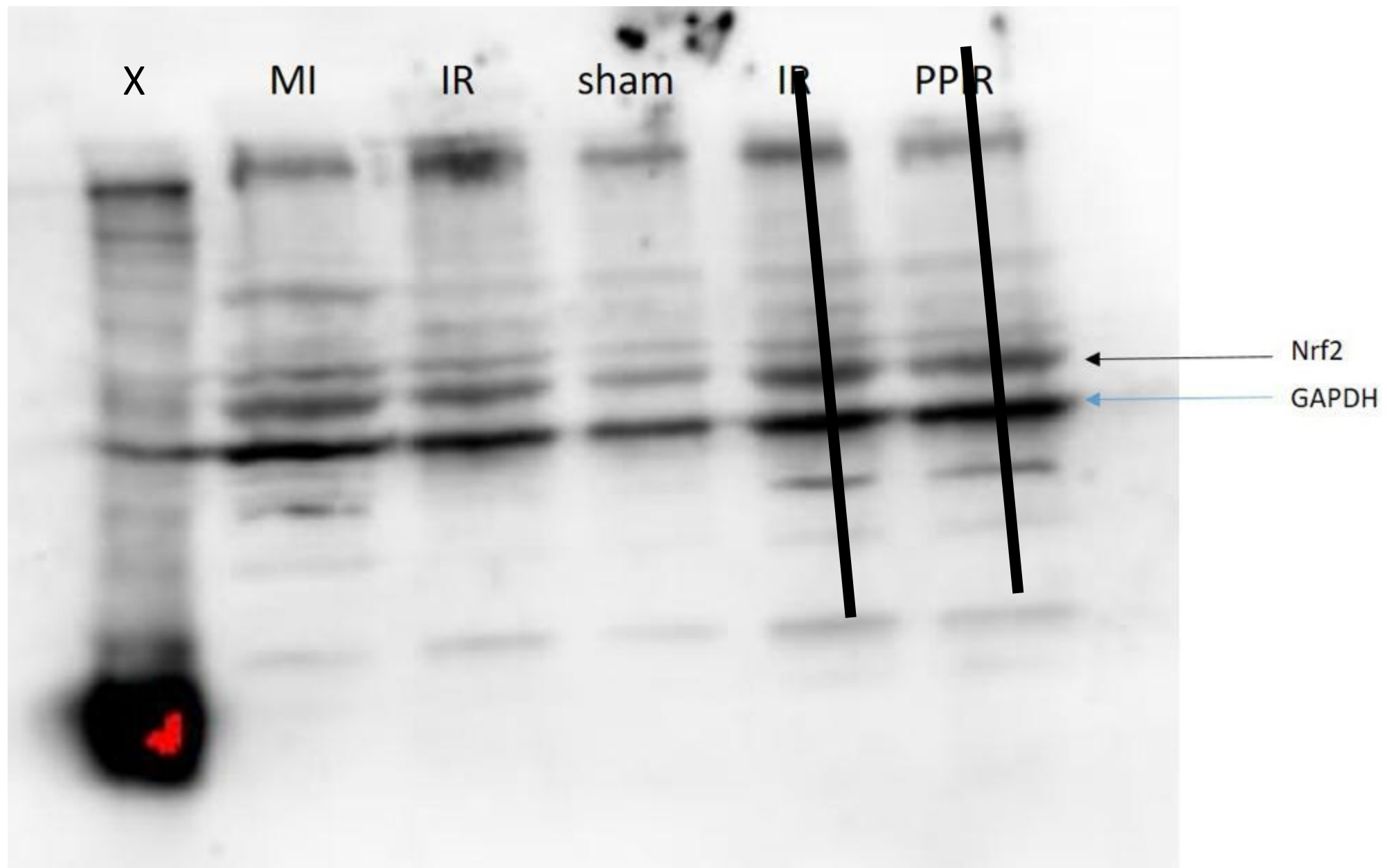

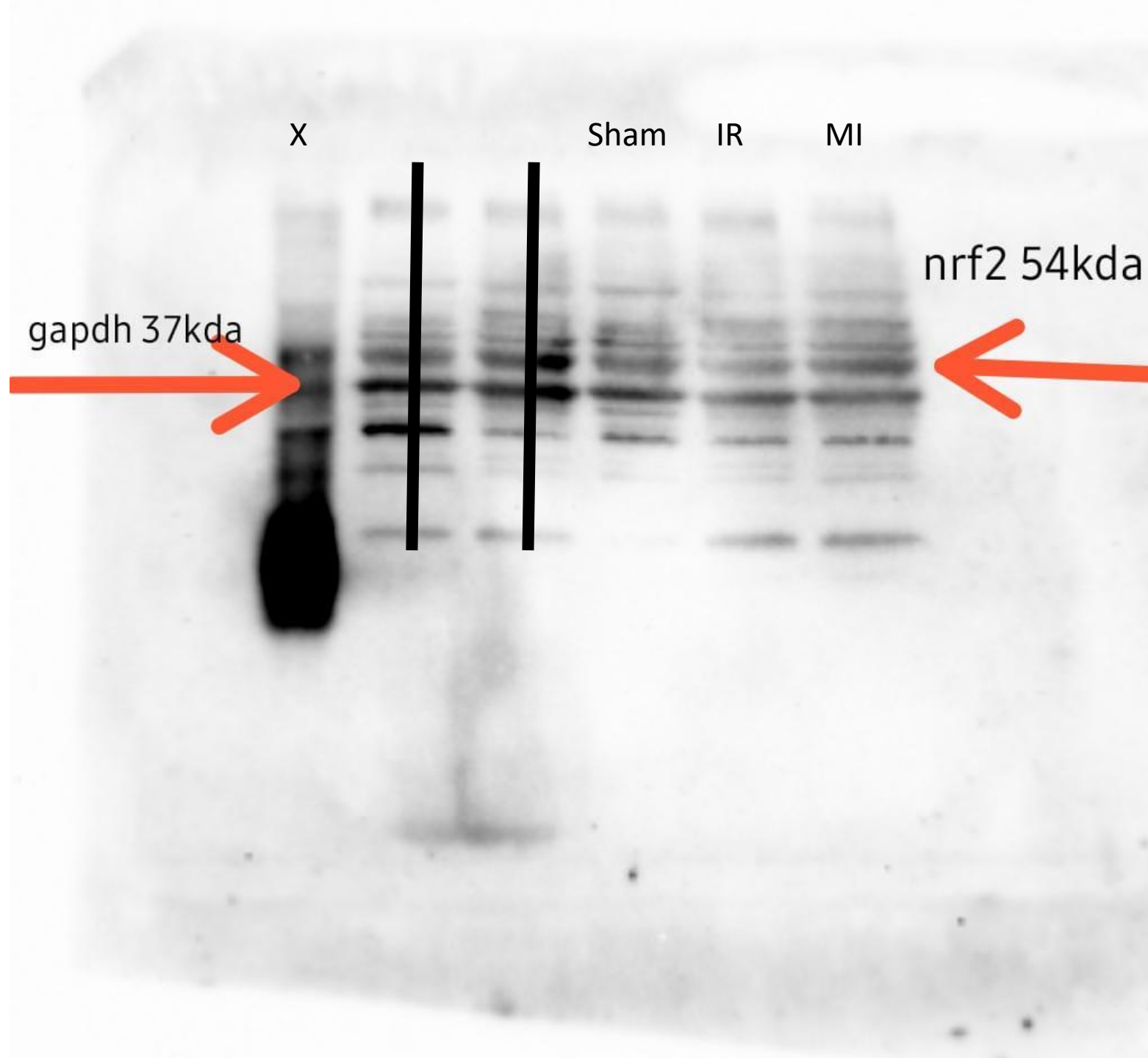

Supplement: S1 Raw images — (PDF) [file pone.0299503.s002.pdf]
